# What is the Impact of Dopamine D2 Receptor in the Brain-Gut Axis? A Narrative Review of the Mechanism Based on Gut Microbiota in Modulating Emotion and Behavior

**DOI:** 10.31083/AP39226

**Published:** 2025-11-19

**Authors:** Ying-Ao Xie, Jian-Da Kong, Shuai Li, Dian-Fang Wei

**Affiliations:** ^1^School of Basic Medicine, Qilu Medical University, 255314 Zibo, Shandong, China; ^2^Shool of Clinical Medicine, Jining Medical University, 255314 Jining, Shandong, China; ^3^Department of Physical Education, Qufu Normal University, 273165 Qufu, Shandong, China

**Keywords:** dopamine, D2 receptor, GBA, gut microbiota, emotion, behavior, mechanism

## Abstract

With the development of the brain-gut axis (GBA), the bidirectional communication between gut microbiota and the brain has become critical in emotion regulation research, and dopamine D2 receptors and gut microbiota play key roles in this process, especially in neurological and psychiatric disorders. This narrative review explores the impact of dopamine D2 receptors in the GBA, focusing on how gut microbiota modulates emotion and behavior via D2 receptors, analyzes their imbalance correlation, and looks forward to D2 receptor-based therapies.Comprehensive searches were conducted in PubMed, Web of Science, and Google Scholar (2000-2025) using keywords like “dopamine D2 receptor”, “brain-gut axis”, and “emotional disorders”, including animal and clinical studies. Research shows gut microbiota affects dopamine system activity and D2 receptor function mainly via metabolites, especially short-chain fatty acids (SCFAs, such as butyric acid and propionic acid). SCFAs cross the blood-brain barrier, bind to G protein-coupled receptors (GPCRs) to regulate dopamine synthesis/release, enhance brain immune function by improving astrocyte activity and blood-brain barrier integrity, and thus promote D2 receptor signal transduction. Gut microbiota also indirectly influences D2 receptor expression/activity by regulating dopamine precursor (such as tyrosine) metabolism. Gut microbiota imbalance is closely associated with D2 receptor dysfunction. In depression, anxiety, schizophrenia, and Parkinson’s disease, D2 receptor function is reduced or abnormally activated; gut dysbiosis (such as *altered Firmicutes*/*Bacteroidetes ratio*, increased *Proteobacteria*/*Escherichia coli*) disrupts gut metabolites (such as reduced SCFAs), aggravates systemic inflammation, and impairs the dopamine system. Overall, gut microbiota modulates D2 receptor activity through multiple mechanisms, exerting an important role in regulating emotion and behavior.

## Main Points

1. This review clarifies the core role of dopamine D2 receptors in the brain-gut axis (GBA), emphasizing their significance in mediating bidirectional communication between the gut microbiota and the brain for emotion and behavior regulation.

2. Key mechanisms by which gut microbiota modulates D2 receptor function are identified: short-chain fatty acids (SCFAs, such as butyric acid, propionic acid) cross the blood-brain barrier, bind to G protein-coupled receptors (GPCRs) to regulate dopamine synthesis/release, and enhance D2 receptor signal transduction by improving brain immune function (such as astrocyte activity, blood-brain barrier integrity); gut microbiota also indirectly affects D2 receptor expression/activity via regulating dopamine precursor (such as tyrosine) metabolism.

3. A close correlation between gut microbiota imbalance and D2 receptor dysfunction is confirmed: in neuropsychiatric disorders (such as depression, anxiety and schizophrenia) and neurodegenerative diseases (Parkinson’s disease), gut dysbiosis (such as altered Firmicutes/Bacteroidetes ratio, increased Proteobacteria/Escherichia coli) disrupts metabolites (such as reduced SCFAs), exacerbates systemic inflammation, and impairs D2 receptor function, contributing to symptom progression.

4. This review proposes potential therapeutic directions: targeting D2 receptors combined with gut microbiota modulation (such as probiotics, dietary adjustments) may provide new strategies for managing mood disorders and other GBA-related neurological/psychiatric conditions.

## 1. Introduction

In the gut–brain axis (GBA), the bidirectional communication mechanism between the gut and the brain has commonly drawn extensive attention [1]. The GBA signifies the complex regulatory network formed via multiple pathways, such as neural, endocrine, and immune, which not only influences digestive function, but also is crucial for emotion, behavior, and cognitive mechanisms [2, 3]. Currently, evidence has been reported that the impact of gut microbiota on modulating brain function and emotional state should not be overlooked, in particular, in the pathogenesis of neuropsychiatric disorders, including mood disorders, anxiety, and depression, the correlation between changes in gut microbiota and neural activity has been commonly documented [2, 4].

Among the most notable of these, the role of the dopamine system in emotion regulation has been broadly determined. As a major neurotransmitter in the brain, dopamine takes part in the regulation of emotion, motivation, learning, and behavior via its different types of receptors (especially D2 receptor) [5, 6, 7]. For instance, dopamine is crucial for modulating emotion, reward, and satiety, and gut microbiota is tightly linked to the bioavailability of dopamine via the microbiota-brain-gut axis, which indicates that gut microbiota, not only influences the synthesis and metabolism of dopamine in the context of modulating the nervous system, immune system, and intestinal metabolites, but also influences the brain’s emotion and behavior [8]. Furthermore, it is apparent that the gut microbiota interacts mutually with the brain via the vagus nerve and influences the dopamine concentration in the brain, in detail, the impact of metabolites, such as short-chain fatty acids (SCFAs), has a vital implication for the brain’s emotion regulation [8]. Thus, these microorganisms with metabolites play a vital role in the regulation of the dopamine system, and thus impact behavior and emotion [8]. However, it continues to be obscure what the exact interaction mechanisms are among gut microbiota, D2 receptor, and emotion regulation.

Consequently, this review is designed to delve into the impact of the dopamine D2 receptor in the GBA, deeply investigate how gut microbiota modulates emotion and behavior via this receptor, and assess its potential implications for neuropsychiatric disorders, such as mood disorders. Based on current research achievements, we also aspire to offer new perspectives and directions for future scholarly work in Dopamine D2 Receptor-related realms.

## 2. Methodology

### 2.1 Search Strategy

A comprehensive search was performed across multiple academic databases, including PubMed (https://pubmed.ncbi.nlm.nih.gov), Web of Science (https://www.webofscience.com), and Google Scholar (https://scholar.google.com). The search covered the period from January 2020 to March 2025, to capture the most recent and relevant studies. Keywords used in the search included “Dopamine D2 receptor”, “Brain-gut axis”, “Gut microbiota”, “Emotion regulation”, and “Mood disorders”, among others. Boolean operators (AND, OR) were used to combine these keywords and ensure the broadest possible coverage of the literature.

### 2.2 Inclusion and Exclusion Criteria

The inclusion criteria for selecting studies involved peer-reviewed publications that were published from 2000 onward; in detail, those that investigated the correlation between the dopamine D2 receptor and gut microbiota. Studies addressing the regulatory effects of gut microbiota on emotion and behavior, in particular, in mood disorders, such as depression, anxiety, and schizophrenia, were included. Animal and human studies were both considered. Conversely, studies not directly linked to the GBA or dopamine receptor function, and non-English language papers, were excluded. Editorials, opinions, and non-original research articles were also excluded from the review.

### 2.3 Study Selection Process

The study selection process followed a rigorous multi-step procedure to ensure relevance and quality. Initially, a broad search was conducted using the predefined keywords. The titles and abstracts of the retrieved papers were screened for relevance. Those that met the inclusion criteria were then subjected to a full-text review. After carefully evaluating the full-text articles, relevant data, including study design, methods, and key findings, were extracted for further analysis. This process ensured that only the most pertinent studies were included in the review.

### 2.4 Study Selection Process

To visually represent the study selection process, the following process was utilized: Initial Search: A broad search across selected databases. Screening of Titles and Abstracts: The first filter based on relevance to the topic. Full-Text Review: Evaluation of studies based on the inclusion/exclusion criteria. Data Extraction: Extraction of relevant data from eligible studies. Synthesis: Categorization and integration of data into the review’s narrative. This process ensured a comprehensive selection of studies, focusing on key areas like dopamine D2 receptor function, the impact of gut microbiota, and their implications for mood disorders.

### 2.5 Analysis and Reporting

The selected studies were analyzed to identify key mechanisms in the context of which gut microbiota influences dopamine receptor function, in particular, the D2 receptor. Special attention was given to how alterations in gut microbiota contribute to mood disorders, such as depression, anxiety, and schizophrenia, in the context of interrupting dopamine signaling. The findings from animal models and clinical studies were integrated, highlighting experimental evidence and clinical implications. This narrative review synthesizes findings to offer insights into the bidirectional communication between the gut microbiota and the dopamine system, offering new perspectives on potential therapeutic strategies for mood disorders.

## 3. Results

### 3.1 Basic Concepts and Roles of the GBA

The GBA holds significance within the domain of biological research, with its intricate network including different physiological and pathological mechanisms [9]. To obtain a in-depth understanding of this system, the crux lies in determining its definition and bidirectional regulatory mechanism. It is worth noting that the gut microbiota plays a vital role in the GBA, and its implications on brain function serve as one of the focal points in this research area. In the following sections, we will delve into these perspectives in detail.

#### 3.1.1 Definition and Bidirectional Regulatory Mechanism of the GBA

The GBA signifies the bidirectional interconnected system. This gives rise to a regulatory system via the complex interaction of the nervous, endocrine, and immune systems, including an extensive scope of physiological and pathological mechanisms. Currently, research on the GBA has commonly drawn comprehensive attention in the academic community, specifically regarding the interaction between the nervous system, immune system, and gut microbiota [2]. The central mechanism of the GBA counts on the interaction. The gut not only functions as a digestive organ, but is also regarded as a vital regulatory factor impacting brain function, emotion, and behavior [10].

From the aspect of neurobiology, the GBA establishes a direct connection via the vagus nerve, forming a neural signal transmission path. The vagus nerve is a key part of the GBA. It not only carries information from the gut to the brain, but also allows the brain to reversely modulate the function of the gut via this nerve [11]. Besides, the signal transmission in the GBA also entails the integrated action of endocrine pathways (such as gastrointestinal hormones), immune pathways (such as cytokines), and metabolites (SCFAs), which jointly take part in the regulation of these interactions [3, 12].

During these interactions, the metabolites of gut microbiota step into the brain via the bloodstream and modulate the function of the nervous system. For instance, SCFAs, like butyric acid and propionic acid, synthesized by gut microbiota, can cross the blood-brain barrier and impact the neural activity of the brain, thus playing a vital role in the regulation of emotion, cognition, and behavior [3, 13]. Furthermore, about 70% of the immune cells in the body are centered in the gut. Thus, these immune cells perform immune defense function locally in the gut, and communicate with the immune system of the brain via inflammatory mediators (such as cytokines), hence impacting brain function and health [3, 13].

The bidirectional regulatory property is one of the central features of the GBA. Articles have reported that the bidirectional communication of the brain-gut-immune axis is crucial for sustaining the balance of the body. It is worth noting that gut microbiota can impact brain function via immune and neuroendocrine pathways [3, 14]. For instance, El Aidy *et al*. [15] reported that gut microbiota can communicate with the brain via the vagus nerve, impact neurotransmitters and immune signals, thus modulating the physiological state of the brain. Furthermore, the imbalance of gut microbiota (such as gut dysbiosis) is tightly linked to different neuroinflammatory diseases [12]. This bidirectional regulatory mechanism is displayed in the impact of changes in gut microbiota on brain function, regulation of emotion, behavior, and cognitive function [13]. Hence, the composition of gut microbiota and its metabolites may play a vital role in the occurrence and development of different neuropsychiatric disorders, thereby influencing emotional, behavioral, and cognitive processes [3].

Generally, the bidirectional regulatory mechanism of the GBA reflects the complex correlation reflects the complex correlation, and offers a new perspective for research on emotion, behavior, and cognitive function. In light of the role of the GBA, research in this field is expected to offer new ideas and methods for the prevention and treatment of neuropsychiatric disorders.

#### 3.1.2 The Impact of Gut Microbiota on Brain Function

It is interesting that gut microbiota is crucial for modulating immune responses and maintaining the function of the blood-brain barrier via its metabolites, in detail, SCFAs. For instance, a study by Silva *et al*. [13] reported that these metabolites step into the brain via the bloodstream and can directly or indirectly impact the inflammatory response and neuroimmune environment of the brain. Specifically, gut microbiota can modulate the immune state of the brain via its microbial metabolic activities, thus playing a vital role in sustaining the integrity of the blood-brain barrier [13]. Hence, gut microbiota not only influences immune responses, but also is crucial for the immune regulation of the brain via microbial metabolites. Furthermore, it is worth noting that gut microbiota can further impact brain function in the context of impacting the synthesis and metabolism of neurotransmitters. For instance, gut microbiota can change the level of serotonin in the brain in the context of modulating the metabolic pathway of tryptophan, thus having vital implications for emotion, anxiety, and cognitive function [16]. This process determines that gut microbiota continues to participate in immune regulation, and at the same time, directly influences the emotional and cognitive mechanisms of the brain at the neurobiological level, in particular, the regulation of dopamine synthesis and receptor activity by gut microbiota further heightens its role in emotion and behavior regulation [8].

The impact of gut microbiota is not limited to directly modulating the level of neurotransmitters, and also modulates the systemic immune response via its interaction with the gut immune system. This process is especially vital in light of psychiatric disorders. Furthermore, the activities of immune cells and cytokines impact gut function, are transported to the brain via the bloodstream, modulate neuroimmune responses, and thus impact the physiological state of the brain [17, 18]. In particular, regarding gut microbiota imbalance, the occurrence of systemic inflammatory responses not only influences gut function, but also acts on the brain via immune-mediated pathways, thus modifying the emotion and behavior [12].

Through these mechanisms, gut microbiota can impact the function of the brain via multiple pathways, and play a key role in modulating emotion and cognitive function. In particular, in light of mood disorders, such as depression and anxiety, changes in gut microbiota may be crucial for brain dysfunction. Thus, the interaction between gut microbiota and the brain not only offers a new biological perspective, but also provides a theoretical basis for the development of new treatment strategies for psychiatric disorders.

### 3.2 The Impact of Dopamine D2 Receptor in Emotion Regulation

**Fig. 1.**
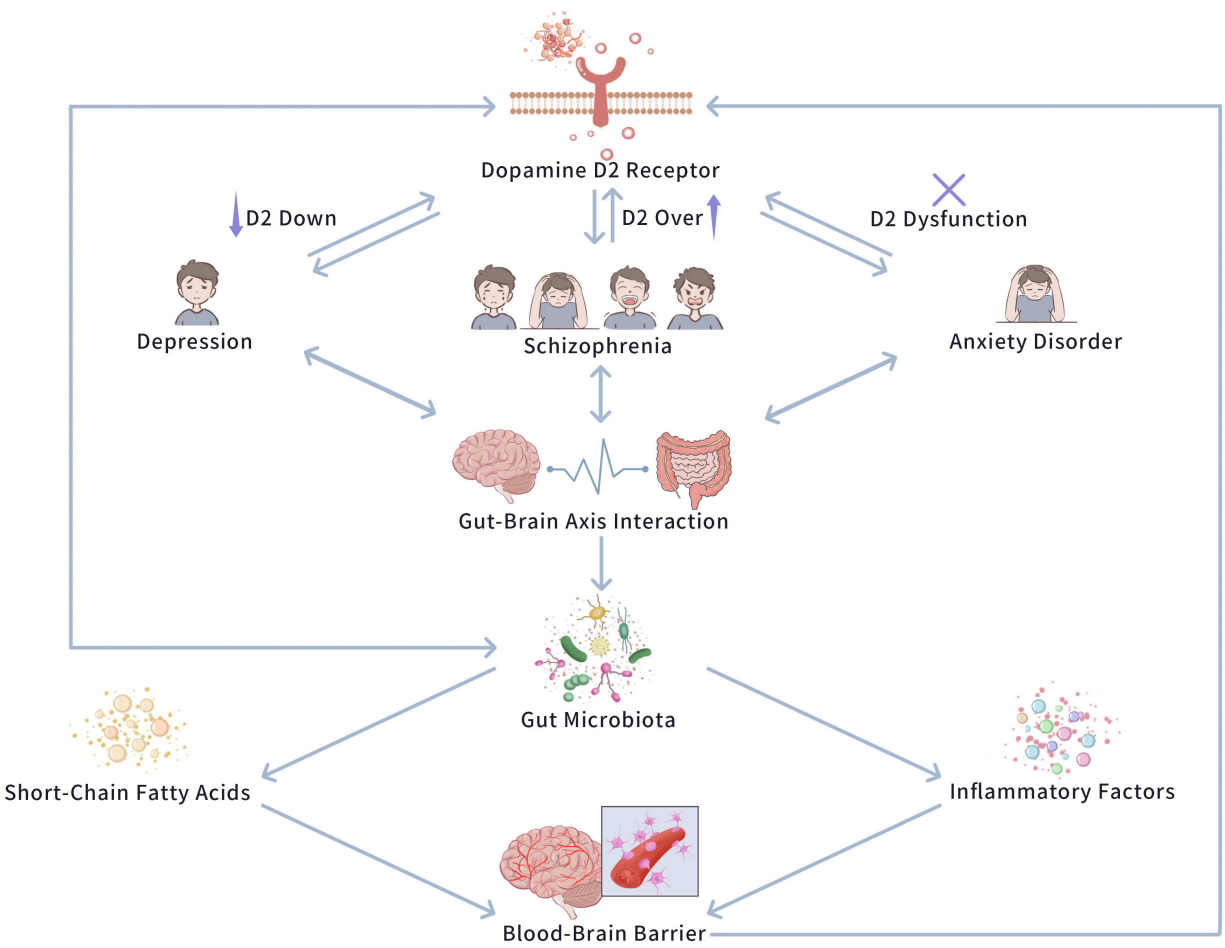
**The central role of Dopamine D2 Receptor in emotional regulation and psychiatric disorders**. This figure exhibits the central position of the dopamine D2 receptor in modulating emotional responses and its pathological relevance in major psychiatric disorders, including depression, anxiety, and schizophrenia. The D2 receptor regulates dopaminergic transmission, and its dysfunction causes mood disturbances. Additionally, the figure emphasizes the role of gut microbiota in influencing D2 receptor function via the brain-gut axis (GBA). Alterations in microbial composition—such as reductions in beneficial species and increases in harmful taxa—disrupt neurotransmitter synthesis and enhance neuroinflammation, which jointly affect D2 receptor activity and contribute to emotional and behavioral dysregulation. The figure was created using Adobe Photoshop (version 25.0, Adobe Inc., San Jose, CA, USA).

As a vital part of the neurotransmitter system, the dopamine D2 receptor plays a key role in emotion regulation. In the following sections, we will further delve into its status within the neurotransmitter system and the intimate associations it has with mood disorders. Fig. 1 exhibits the central impact of Dopamine D2 Receptor in emotion regulation.

#### 3.2.1 The Status of D2 Receptor in the Neurotransmitter System

The dopamine system is central to emotion and behavior regulation, in detail, the D2 receptor, which is crucial for dopaminergic signal transduction [7]. The D2 receptor is part of the dopamine receptor family, and is broadly distributed in the central nervous system (CNS), in particular in brain regions linked to emotion, reward, cognition, and movement, including the nucleus accumbens, striatum, and prefrontal cortex [19]. The D2 receptor, via its self-inhibitory effect, serves as a central component of the negative feedback mechanism, modulating the release and reuptake of neurotransmitters and sustaining the normal function of the dopamine neural pathway [20]. Hence, the normal function of the D2 receptor is vital for emotional stability. In this regard, an article has displayed that the D2 receptor inhibits the excessive release of dopamine via negative feedback regulation to avoid overstimulation of the nervous system [21]. When the dopamine system is dysregulated, particularly when the activity of the D2 receptor is compromised, mood disorders frequently arise [22]. The overstimulation of the D2 receptor may result in “dopamine supersensitivity”, which is especially ubiquitous in some schizophrenia patients who have received chronic antipsychotic therapy [21].

#### 3.2.2 The Correlation Between D2 Receptor and Mood Disorders

In light of mood disorders, the dysfunction of the D2 receptor is tightly linked to different psychiatric disorders. Studies have reported that patients with depression generally display a reduction in the expression of D2 receptors, and it is evident that this change may weaken the reactivity of the brain’s reward system, thus eliciting symptoms, including low mood and lack of motivation [7, 23]. Moreover, the alterations in the activity of D2 receptors in patients with anxiety also suggest that the dopamine system is crucial for the stress response to environmental threats [24, 25]. The change in this regulatory capacity may give rise to the emergence of anxiety symptoms [7, 26]. Furthermore, the dysfunction of D2 receptors in patients with schizophrenia is particularly vital. For instance, articles have determined that the hyperactivation of the dopamine system is directly linked to the emergence of psychotic symptoms, such as hallucinations and delusions. Hence, the D2 receptor turns into the main target of antipsychotic drugs [27, 28].

### 3.3 The Interaction Between Gut Microbiota and D2 Receptor

After determining the key impact of the dopamine D2 receptor in emotion regulation, we will next delve into the critical aspect of the crosstalk between the gut microbiota and the D2 receptor [8, 29]. Among these most notable of these, the regulatory mechanisms through which the gut microbiota modulates the function of the D2 receptor and the exact mechanisms by which the D2 receptor is involved in the GBA are central to gaining a key understanding of their correlation between them. We will analyze these in detail below [8, 30].

**Fig. 2.**
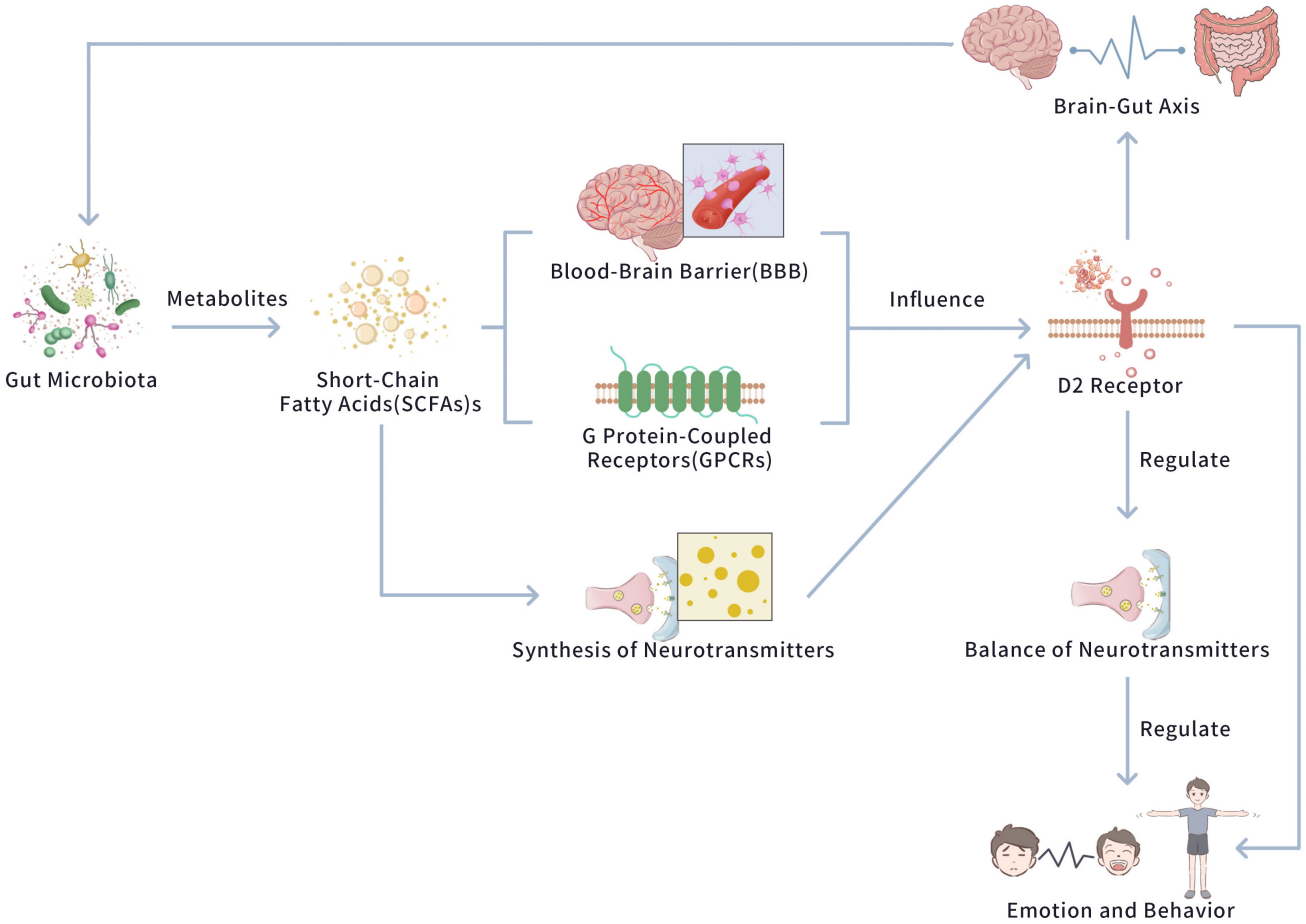
**The interaction between gut microbiota and D2 receptor**. This figure exhibits how gut microbiota influences brain function via the secretion of metabolites, such as short-chain fatty acids (SCFAs). These metabolites act on the blood-brain barrier (BBB) and G protein-coupled receptors (GPCRs), influencing neurotransmitter synthesis. This cascade of events ultimately modulates the D2 receptor, a key regulator in maintaining the balance of neurotransmitters. The regulation of the D2 receptor then governs emotional responses and behavioral outcomes, highlighting the bidirectional communication within the GBA. The figure was created using Adobe Photoshop.

The crosstalk between gut microbiota and the dopamine D2 receptor emerges as a key research direction. Specifically, through a range of metabolic pathways, gut microbiota can directly or indirectly modulate the function of the D2 receptor [8, 13]. Studies have revealed that gut microbiota, via its metabolites, in particular SCFAs, has vital implications for the activity of the dopamine system in the brain, in detail, the D2 receptor [8, 13]. Fig. 2 exhibits the interaction between Gut Microbiota and D2 Receptor.

#### 3.3.1 How Gut Microbiota Modulates the Function of D2 Receptor

Current evidence has reported that gut microbiota influences the function of the D2 receptor via its metabolites, in particular SCFAs, such as butyric acid and propionic acid [8, 31]. For instance, these SCFAs can not only traverse the blood-brain barrier (BBB), but also bind to GPCRs in the brain to promote signal transmission, hence influencing different physiological mechanisms [13]. Specifically, SCFAs, such as butyric acid and propionic acid, have been reported to enhance the integrity of the blood-brain barrier in the context of modulating the function of astrocytes, which are crucial in forming and maintaining the barrier. Additionally, these metabolites modulate brain inflammatory responses in the context of impacting microglial cells, which play a key role in immune surveillance and neuroinflammation [32]. This interaction not only modulates the function of neurons and astrocytes, but also vitally influences emotion and behavior.

In addition, the potentiating effect of SCFAs on D2 receptor signal transduction further determines the regulatory impact of gut microbiota on emotion and behavior processes [8, 33]. In the context of acting on neurotransmitter synthesis, in particular dopamine, gut microbiota can significantly impact the function of the D2 receptor [32]. Gut microbiota indirectly influences the expression and activity of the D2 receptor in the context of modulating the metabolism of dopamine precursor molecules (such as tyrosine), providing a mechanistic basis for the impact of gut microbiota on emotion regulation [8].

In addition to dopamine transporter (DAT), other mechanisms also participate in the crosstalk between gut microbiota and the dopamine system [8]. For instance, research has reported that metabolites synthesized by specific gut bacteria, including indoles and phenolic compounds, can impact the expression and activity of dopamine D2 receptors. Notably, Scott *et al*. (2024) [34] uncovered that tryptophan metabolites produced by enteric bacteria counteract the invasion of intestinal pathogens by activating dopamine D2 receptors in the intestinal epithelium. Furthermore, Tennoune *et al*. (2022) [35] pointed out that indole compounds produced by the gut microbiota, such as indole and indole sulfonic acid, can cross into the brain via as-yet-unidentified mechanisms and affect neural activity and emotional behavior at varying doses, indicating that these metabolites play a crucial role in modulating neurotransmitter activity. These metabolites bind to GPCRs on the brain neurons, indirectly modulating dopamine receptor activity and influencing the neuroplasticity essential for emotion and behavior regulation [36].

Furthermore, gut microbiota can also modulate the biosynthesis pathways of dopamine precursors. For instance, gut bacteria may impact the metabolism of tyrosine, the amino acid precursor to dopamine, in the context of modulating gut-derived enzymes and metabolic pathways; intriguingly, this regulation of dopamine synthesis further influences the activity of the D2 receptor, enhancing or inhibiting its function based on based on dopamine levels in the synaptic cleft [8, 36]. Accumulating evidence indicates that the gut-brain axis plays a vital role in maintaining appropriate dopamine levels via sophisticated crosstalk between the gut microbiota and the brain. Microbial populations in the gut can directly impact the synthesis of neurotransmitters, including dopamine, hence impacting neurochemical signaling in the brain. Additionally, interruptions in gut microbiota composition may be linked to neurodegenerative diseases, such as Parkinson’s disease, by impairing dopaminergic function [8, 36].

Thus, the crosstalk between gut microbiota and the D2 receptor is multidimensional, including not only SCFAs but also other metabolites, as well as regulatory mechanisms such as DAT modulation and changes in dopamine precursor biosynthesis. These mechanisms, via their implications for dopamine signaling, play a vital role in regulating mood, behavior, and cognitive function.

#### 3.3.2 The Mechanism of D2 Receptor in the GBA

As a critical hub in dopamine signal transduction, the D2 receptor plays a key role in the nervous system, and co-modulates neurotransmitter homeostasis in the brain with the metabolites of gut microbiota (such as SCFAs) [8, 37, 38]. The regulatory effect of gut microbiota metabolites on the D2 receptor can indirectly impact the regulation of emotion and behavior, and this mechanism is shaped by the impact of microbial metabolites on the immune and endocrine pathways [8].

Additionally, research has reported that the gut microbiota directly modulates key neurotransmitters including dopamine, serotonin, and glutamate via bidirectional crosstalk with the brain, which are crucial for emotion regulation. The gut microbiota influences emotional responses and behavior by influencing the levels of these neurotransmitters. For instance, some gut bacteria can synthesize neurotransmitters, such as dopamine, which not only act locally in the gut, but also impact brain function, hence modulating emotions and cognitive states [8]. Furthermore, metabolites generated by the gut microbiota also have a regulatory effect on the physiological functions and behaviors of the CNS and the enteric nervous system (ENS), suggesting that the gut microbiota influences mood and behavior by modulating neurotransmitters [39]. Precisely, studies have reported that the gut microbiota modulates emotions and behavior by influencing neural circuits and neurotransmitter expression. For instance, a study reports that the gut microbiota can impact the brain dopamine pathway linked to eating behavior, in particular, by modifying the expression of dopamine D1 and D2 receptors, hence impacting impulsive behavior and reward responses [40]. Notably, the composition of the microbiota and the levels of SCFAs are closely associated with human short-term memory and working memory, indicating that the gut microbiota is crucial for modulating emotions and behavior by affecting the dopamine system’s response to external stimuli [40]. These studies indicate that the gut microbiota not only influences the function of dopamine receptors, but also is tightly linked to the brain’s response to external stimuli by modulating neural circuits, ultimately playing an important role in modulating emotions and behavior [41, 42]. It is interesting that the microbiota interacts with the brain via multiple pathways, such as the vagus nerve, immune system, and hypothalamic-pituitary-adrenal axis, thus regulating emotional and behavioral processes [43]. In this process, the dysfunction of the D2 receptor, specifically, the abnormality in signal transduction, may emerge as a key factor in the occurrence of mood disorders triggered by gut microbiota imbalance, thus holding critical implications for the behavior and emotional state of individuals [44].

### 3.4 The Impact of D2 Receptor and Gut Microbiota Imbalance in Mood Disorders

**Fig. 3.**
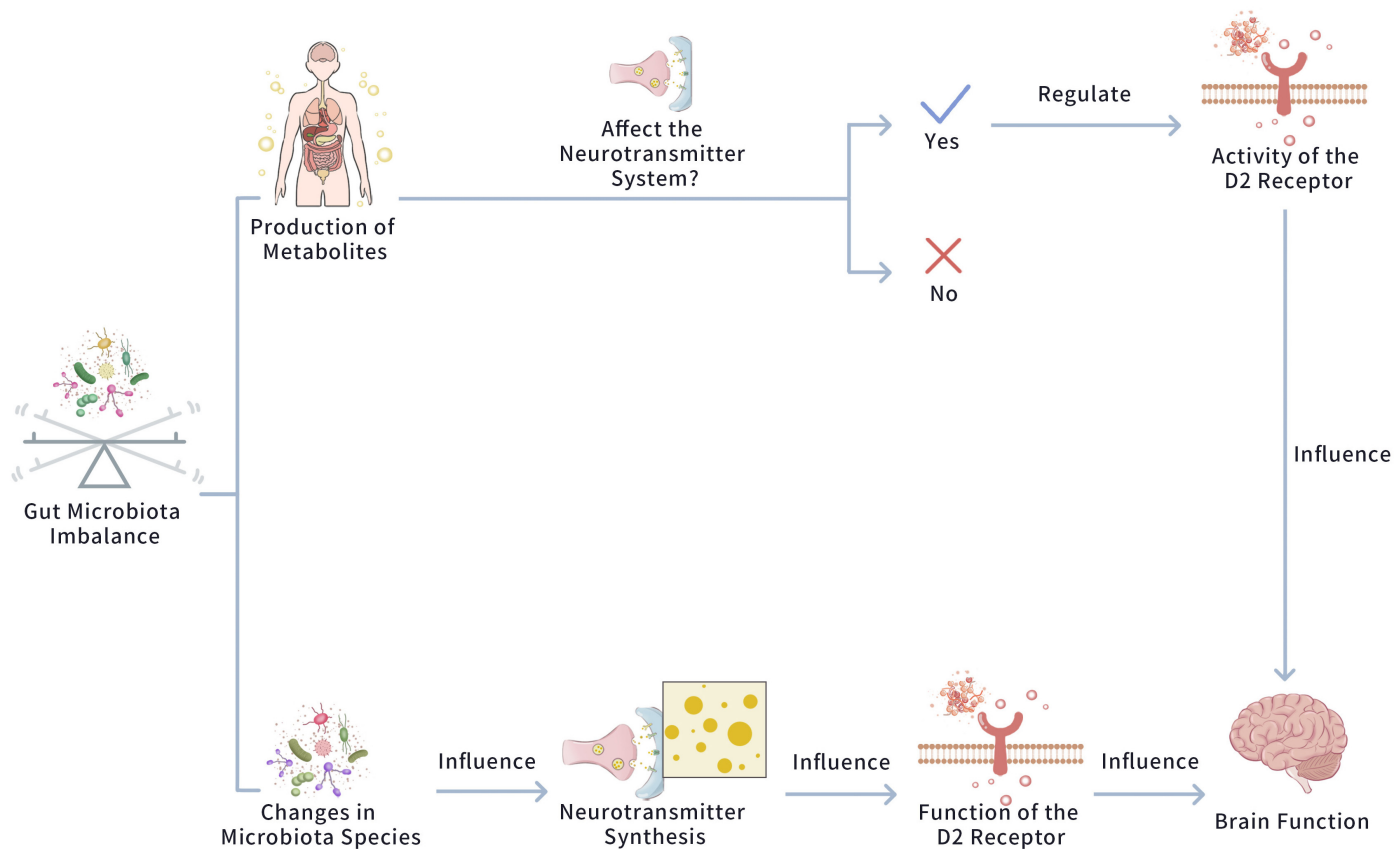
**The impact of D2 receptor and gut microbiota imbalance in mood disorders**. This figure exhibits how a gut microbiota dysbiosis triggers changes in microbiota species, which subsequently disrupts neurotransmitter synthesis. This interruption then influences the activity of the D2 receptor, and the altered function of the D2 receptor impairs overall brain function.

**Fig. 4.**
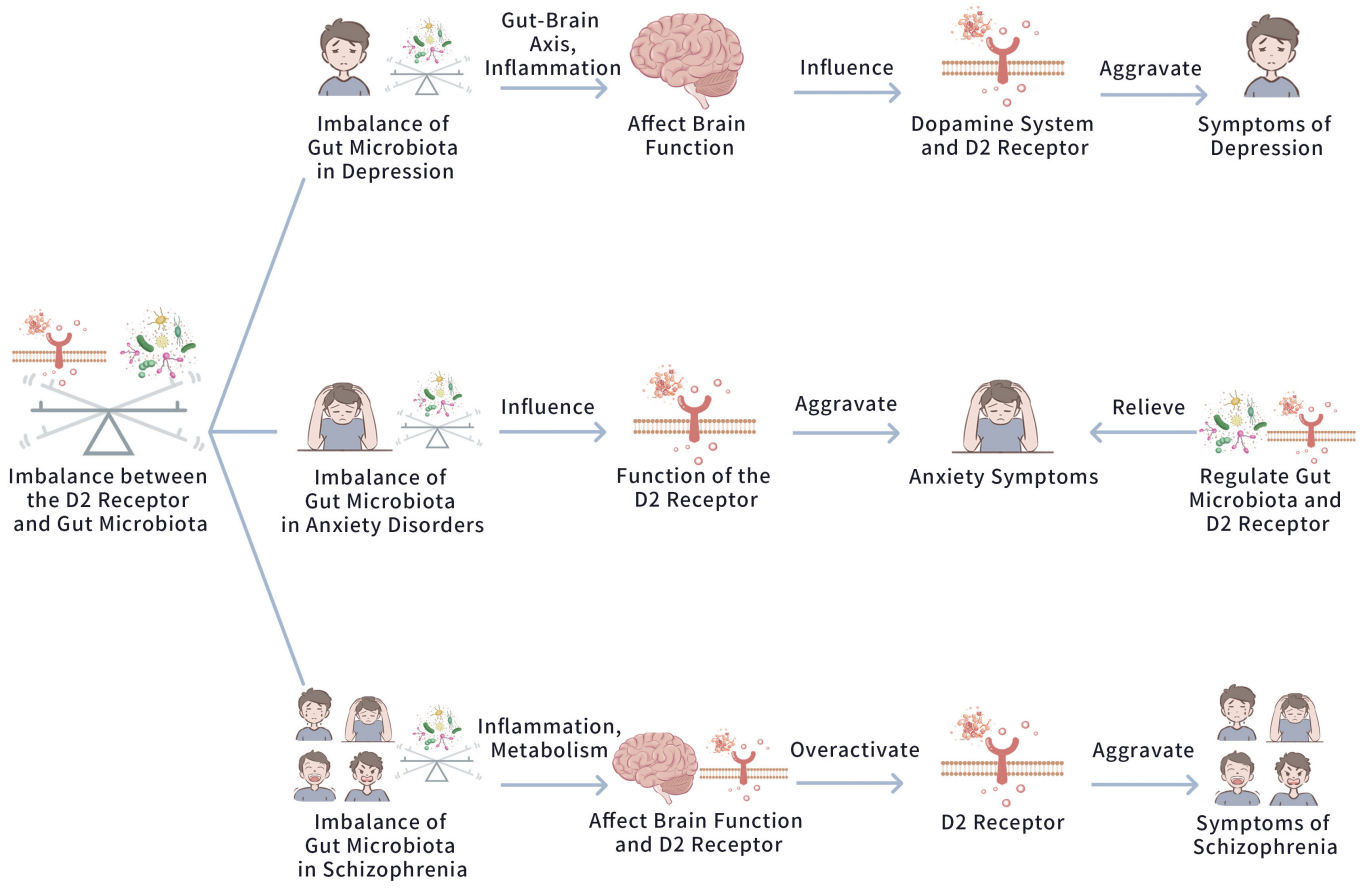
**The impact of D2 receptor and gut microbiota imbalance in mood disorders**. This figure illustrates how gut microbiota dysbiosis influences psychiatric conditions by modulating the brain’s neurotransmitter systems. In depression, a gut microbiota dysbiosis triggers gut-brain axis inflammation, which impairs brain function and exacerbates dopamine system dysfunction, hence worsening depressive symptoms. Similarly, in anxiety disorders, gut microbiota imbalance influences the D2 receptor’s function, aggravating anxiety symptoms. In schizophrenia, both inflammation and metabolic disturbances arising from gut microbiota imbalance alter neural activity and D2 receptor activity, triggering exacerbated symptoms severity. Crucially, modulating the gut microbiota and D2 receptor can help alleviate the symptoms of these conditions, suggesting potential therapeutic pathways for controlling these psychiatric disorders by targeting the gut-brain axis.

Considering the intimate association between the gut microbiota and the D2 receptor, we further explore their roles in mood disorders [45, 46, 47]. Specifically, the correlation between gut microbiota dysbiosis and D2 receptor dysfunction, and the exact interactions between these two factors in mood disorders, are critical for understanding the pathogenesis of mood disorders [45, 48]. In the subsequent sections, we will perform an in-depth analysis of these perspectives. Figs. 3,4 exhibit the impact of D2 Receptor and Gut Microbiota Imbalance in Mood Disorders [8, 47, 49].

#### 3.4.1 The Correlation Between Gut Microbiota Imbalance and D2 Receptor Dysfunction

The diversity and homeostasis of gut microbiota play a vital role in emotion regulation; specifically, holding significant implications for the brain’s emotion regulation function [46, 50]. Notably, recent research has reported that gut microbiota imbalance (such as reduced microbiota diversity and specific microbiota imbalance) is tightly linked to different neuropsychiatric disorders (such as depression, anxiety, and schizophrenia) [51]. This determines that gut microbiota dysbiosis not only influences the physiological function of the gut, but also holds significant implications for brain function via the GBA [49, 52]. The dysregulation of the dopamine system triggered by gut microbiota imbalance is strongly linked to the development of mood disorders [53].

3.4.1.1 The Association Between Reduced Microbiota Diversity and D2 Receptor Function

Decline in gut microbiota diversity is commonly accompanied by the overgrowth of harmful microbiota, and the reduced abundance of probiotics may result in the imbalance of neuro-immune regulation [54, 55, 56]. Probiotics play a vital role in sustaining the healthy homeostasis of gut microbiota and improving the immune function of the host. In particular, in the context of enhancing the abundance of beneficial bacteria, they can relieve the symptoms of chronic inflammation and metabolic disorders [57, 58]. For instance, evidences has determined that gut microbiota imbalance can impact the dopamine pathway in the brain, and thus modify the function of the D2 receptor, for example, the metabolites of gut microbiota, including SCFAs (such as butyrate and propionate), may impact the brain via modulating the activity of the D2 receptor, ultimately playing a vital role in emotion and behavior [58, 59]. Specifically, SCFAs modulate the dopamine system in the brain via the vagus nerve and immune response, and may further impact neurobehavior in the context of modifying the signal transduction pathway of the D2 receptor [57, 59].

Overall, this mechanism describes how gut microbiota modulates neurotransmitters and the functions of their related receptors in the brain via a complex biological signal network, thus impacting emotional stability and behavioral patterns.

3.4.1.2 The Impact of Exact Microbiota Imbalance on D2 Receptor Function

Dopamine is central to modulating mood, cognition, and behavior, with the D2 receptor being one of the key modulators of dopamine signaling in the brain [60]. Interruptions in dopamine signaling, in particular, via alterations in D2 receptor function, are broadly implicated in different neuropsychiatric disorders, including depression, anxiety, and schizophrenia [60]. The intestinal microbiota, with its diverse ecological network of prokaryotes, fungi, and other microorganisms, is increasingly recognized as a key regulator of dopaminergic receptor signaling, including the D2 receptor [61].

Recent studies have highlighted how gut dysbiosis—an imbalance in the gut microbial community—can impact dopamine signaling pathways, hence impacting brain function [8, 62]. In more detail, certain bacterial taxa have been found to be linked to alterations in D2 receptor function, which is involved in the pathophysiology of psychiatric disorders [63].

*Gut Microbiota and D2 Receptor Function in Depression*

According to literature, SCFAs play an important role in sustaining brain health. Exactly, SCFAs such as butyrate can directly impact the expression of brain-derived neurotrophic factor (BDNF) via the blood-brain barrier, which is vital for neuroplasticity and normal function of the dopamine system [64, 65]. In depression, a gut microbiota dysbiosis has been reported to impact the dopaminergic system, in particular, D2 receptors, which are vital for modulating emotions and emotional behavior. A feature of this dysregulation is changes in the abundance of enteric bacteria (such as *Firmicutes* and *Bacteroidetes*), which play an important role in modulating the expression and function of dopamine receptors in the brain. Research has reported that gut microbiota imbalance influences the development of depressive symptoms via abnormal synaptic pruning of glial cells and signal transduction mediated by complement C3 [66]. Furthermore, the imbalance of gut microbiota is tightly linked to the occurrence of depression, in detail, the correlation between changes in gut bacterial communities (such as *Firmicutes* and *Bacteroidetes*) and emotion regulation has been determined [67]. Specifically, studies have reported that *Firmicutes* are often overrepresented in patients with depression, while *Bacteroidetes* is underrepresented, triggering a gut microbiota dysbiosis and interrupting normal dopamine signaling. The literature reports that the increased abundance of *Firmicutes* is tightly linked to the occurrence of different neurological diseases, which is particularly vital in patients with depression [68]. Imbalanced gut microbiota not only influences neurotransmitter synthesis, but also triggers neuroinflammation [69]. Specifically, in animal models lacking gut microbiota entirely, the occurrence of anxiety-like behavior is tightly linked to the absence of gut microbiota [70]. This further confirms the close correlation between gut microbiota and neurological diseases. Hence, the imbalance of gut microbiota is regarded as an important factor in the pathogenesis of depression.

The effects of *Firmicutes* and *Bacteroidetes* on the dopamine system are mainly mediated by the secretion of SCFAs synthesized by specific gut bacteria, such as butyric acid and propionic acid. Research has reported that SCFAs are the main metabolites produced by gut bacteria via fermentation of dietary fiber and resistant starch; they can cross the blood-brain barrier, bind to GPCRs in the brain, and thus impact the activity of microglia and astrocytes. These cell types play a crucial role in regulating neuroinflammation and supporting dopamine receptor function [71]. Research has reported that reduced SCFAs are tightly linked to brain function degeneration, particularly in patients with depression. This change can decrease BDNF levels, impair synaptic plasticity, and exacerbate emotional regulation deficits [64]. Furthermore, gut microbiota imbalance leads to decreased SCFAs secretion, which contributes to the pathogenesis of depression—further demonstrating the crucial role of SCFAs in emotional regulation and neuroplasticity [65]. Notably, SCFAs such as butyrate can directly impact BDNF expression via the blood-brain barrier, which is vital for neuroplasticity and normal dopamine system function [64, 65].

Furthermore, recent reviews indicate that a high abundance of species of *Lactobacilli* and *Bifidobacteria* in the healthy gut microbiota can alleviate depressive symptoms by improving the function of dopamine receptors and lowering neuroinflammation. These species enhance the synthesis of SCFAs, which play an important role in sustaining neuronal plasticity and emotion regulation, in particular, in protecting dopamine receptor function, in the context of modulating immune responses and improving histone acetylation pathways. Research has reported that SCFAs can modulate dopamine and serotonin levels in the gut via the G protein coupled receptor (GPCR) pathway, which has vital implications emotions and behavior. Furthermore, SCFAs enhance intestinal barrier function, promotes the expression and formation of tight junction proteins, and further supports its important role in immune response and neurological health [13].

*Gut Microbiota and D2 Receptor Function in Anxiety*

Anxiety disorders, like depression, are linked to dysregulated dopamine signaling, in particular, at the D2 receptor [72]. The gut-brain axis plays a vital role in this regulation [73]. The overgrowth of pathogenic bacteria, such as *Proteobacteria*, *Enterobacteriaceae*, and *Escherichia coli* has been reported to increase gut permeability and promote the release of pro-inflammatory cytokines [74]. These cytokines can cross the blood-brain barrier, triggering dysregulated dopamine metabolism and D2 receptor dysfunction in regions of the brain linked to emotion and behavioral regulation, such as the prefrontal cortex and amygdala [75].

Additionally, the *Firmicutes*-to-*Bacteroidetes* ratio has been implicated in anxiety disorders, with a higher proportion of *Firmicutes* often seen in individuals with elevated anxiety [52]. This microbial imbalance can directly impact dopamine receptors by increasing neuroinflammation and downregulating dopamine receptor expression, triggering anxiety symptoms [42].

*Gut Microbiota and D2 Receptor Function in Schizophrenia*

Schizophrenia, a severe neuropsychiatric disorder, has also been reported to be impacted by gut microbiota [76]. The impact of the D2 receptor in schizophrenia is well-established, as dopamine dysregulation is regarded as a central feature of the disorder [77]. Recent studies have reported that gut dysbiosis can contribute to dopamine imbalance, in detail, via the crosstalk between microbial metabolites and dopamine receptors [78, 79]. Specific bacterial species, such as *Prevotella* and *Ruminococcus*, have been found to be vitally reduced in individuals with schizophrenia, and this dysbiosis has been linked to exacerbated symptoms of the disorder, including hallucinations and delusions [80, 81].

Research also reports that *Clostridium* species in the gut may impact the dopamine system by modulating the secretion of gamma-aminobutyric acid (GABA), which interacts with dopamine receptors, including D2 [82]. Furthermore, Bacteroides have been reported to promote dopamine receptor expression in the striatum, a key region involved in reward processing, highlighting the importance of a balanced gut microbiome in maintaining D2 receptor function and dopamine signaling in schizophrenia [83].

3.4.1.3 Gut Microbiota Imbalance Alters the Dopamine Signaling Pathway via the D2 Receptor

Gut microbiota imbalance is frequently accompanied by the aberrant release of microbial metabolites (such as endotoxin, SCFAs, neurotransmitters), and these metabolites can impact brain function via immune and endocrine pathways [84]. Additionally, these metabolites have been determined to directly or indirectly modulate the function of the dopamine system, in particular, the function of the D2 receptor [8, 85]. Gut microbiota imbalance may further interrupt the normal function of the D2 receptor in the context of modifying the synthesis and release of dopamine, causing emotional instability and dysregulated behavioral responses [8].

In addition, the gut microbiota indirectly influences the expression and function of the D2 receptor in the context of modulating the balance of neurotransmitters, such as serotonin, norepinephrine, and GABA in the brain [16]. This bidirectional regulation between the gut microbiota and the brain uncovers the vital impact of the gut microbiota on emotion and behavior regulation and offers a novel avenue for further investigation into the mechanism of neuropsychiatric dysfunction.

#### 3.4.2 The Interaction Between the D2 Receptor and Gut Microbiota in Mood Disorders

The occurrence of mood disorders, specifically psychiatric disorders such as depression and anxiety, is intimately linked to the interaction between the D2 receptor and gut microbiota imbalance [49, 86]. The imbalance of gut microbiota can impact the neurotransmitter signaling mechanisms in the brain via the D2 receptor, hence triggering mood disorders [16]. In particular, the interaction between the D2 receptor and gut microbiota may contribute to the development of mood disorders via the following mechanisms [87].

3.4.2.1 The D2 Receptor and Gut Microbiota Imbalance in Depression

In the context of depression, evidence indicates a close association between gut microbiota imbalance and the occurrence and progression of depression [52, 88]. Patients with depression typically exhibit alterations in gut microbiota, and this imbalance can impact brain function via multiple pathways, specifically neurotransmitter signaling linked to the dopamine system [89].

Gut microbiota imbalance is prevalent among patients with depression. Alterations in gut microbiota composition are regarded as a risk factor for different psychiatric disorders. Particularly in depression, gut microbiota imbalance is tightly linked to changes in emotion, anxiety, stress response, and brain function [90]. Some research has revealed that the gut microbiota engages in bidirectional signaling with the brain via the “GBA”, and influences the function of the CNS [8]. Notably, gut microbiota imbalance may increase intestinal permeability and promote the release of inflammatory factors (such as lipopolysaccharide), hence triggering a systemic inflammatory response and ultimately impacting the neuroinflammatory process in the brain [90, 91]. These inflammatory responses can impact the synthesis and metabolism of neurotransmitters in the brain, consequently impacting emotion regulation and behavior.

The dopamine system in the brain of patients with depression generally exhibits functional abnormalities, specifically in the expression and activity of the D2 receptor [92]. The D2 receptor is regarded as a vital target in depression [93]. Studies has found that patients with depression commonly demonstrate low activity of the D2 receptor [8]. This is tightly correlated with the symptoms of depression (such as low mood, lack of interest), as the D2 receptor is crucial for regulating the reward system, motivation, and emotion [7, 94]. Additionally, gut microbiota imbalance may modulate the dopamine system via diverse pathways. For instance, gut microbiota can impact the synthesis of dopamine in the brain via metabolites (such as SCFAs), and intestinal inflammation may further disturb the synthesis of dopamine and the function of the D2 receptor in the context of modifying the inflammatory environment in the brain [8, 90].

The gut microbiota is crucial for modulating BDNF, which is crucial for neural plasticity, synaptic transmission, and overall brain health [95]. The gut microbiota produces metabolites like SCFAs, specifically butyrate, which act as histone deacetylase inhibitors [96]. This action increases the expression of BDNF, supporting brain functions involved in emotion regulation [97]. Reduced BDNF levels have been tightly linked to the development of depression and other mood disorders [98].

When the gut microbiota becomes imbalanced (known as dysbiosis), there is a reduction in SCFAs secretion, leading to increased neuroinflammation [99, 100]. Inflammatory cytokines produced in the gut can cross the blood-brain barrier and impact dopamine signaling, in particular, in the context of inhibiting dopamine receptors like the D2 receptor [101]. The D2 receptor is crucial for emotion regulation, and its dysfunction exacerbates symptoms of depression and anxiety [102]. Thus, gut dysbiosis indirectly impairs dopamine system function in the context of increasing inflammation and lowering BDNF expression [97].

Therefore, the gut microbiota serves as a key mediator in this process, linking BDNF, dopamine signaling, and neuroinflammation [97]. Restoring intestinal barrier integrity and addressing gut inflammation could potentially help to modulate BDNF levels and dopamine function, offering novel therapeutic avenues for managing mood disorders and other neuropsychiatric conditions [103].

In summary, gut microbiota imbalance is tightly tied to the development of depression. The abnormal dopamine system in the brain of patients with depression, specifically the dysfunction of the D2 receptor, may be intimately linked to alterations in the gut microbiota. Further research should delve into how gut microbiota modulates the dopamine system via multiple mechanisms, and explore novel treatment strategies, such as utilizing probiotics and other interventions to modulate the gut microbiota, thereby providing new ideas and approaches for the treatment of depression.

3.4.2.2 The D2 Receptor and Gut Microbiota Imbalance in Anxiety

Anxiety has also been determined to be tightly linked to gut microbiota imbalance and D2 receptor dysfunction [38, 49, 62]. It is worth noting that the manifestation of anxiety is generally accompanied by changes in the gut microbial community, and the function of the D2 receptor may be suppressed in such circumstances [104]. The proliferation of certain pathogenic bacteria is common in the gut microbiota of patients with anxiety; specifically, changes in the abundance of bacteria linked to the stress response, such as *Firmicutes* and *Bacteroidetes*, and their altered abundance is tightly linked to the aggravation of anxiety [67]. These bacterial changes are tightly correlated with the clinical manifestations of anxiety, in particular, in terms of the transmission of impact via the microbiota-gut-brain axis (microbiota-GBA), revealing the potential impact of gut microbiota on the occurrence of anxiety [104].

3.4.2.3 The Impact of the D2 Receptor in the Regulation of Anxiety in the Context of Gut Microbiota

Studies have reported that the D2 receptor plays a vital role in the brain and is tightly linked to anxiety regulation. The activation of the D2 receptor can impact emotion and behavior by modulating the brain’s neurotransmitter systems, specifically the dopamine system. Specifically, the release of dopamine and the activation of the D2 receptor are crucial for relieving anxiety symptoms [104, 105]. Modulating gut microbiota composition can influence the GBA, thereby modifying the activity of D2 receptors in the brain [106]. An article reported that the diversity and health status of the gut microbiota directly impact the function of the nervous system, in particular, in emotion regulation [106]. Beneficial gut microbiota influences the inflammation level and neurotransmitter balance in the brain via mechanisms such as the synthesis of SCFAs and the regulation of the immune response, which is directly linked to the alleviation of anxiety [105, 107]. The supplementation of probiotics has been proven to help improve the balance of the gut microbiota, hence modulating D2 receptor function and alleviating anxiety symptoms. Specific studies have reported that certain probiotics, such as Lactiplantibacillus plantarum, can vitally improve anxiety-like behaviors in mice in the context of modulating tryptophan metabolism and the gut microbiota structure [105]. A diet rich in fiber and variety (such as one abundant in different vegetables, fruits, and whole grains) also helps enhance the diversity of the gut microbiota, hence improving the symptoms of anxiety [104].

3.4.2.4 The Interaction Between the D2 Receptor and Gut Microbiota in Schizophrenia

Lately, more and more articles have suggested that the correlation between gut microbiota and schizophrenia may serve as a vital pathological basis of the disease [108, 109]. Studies have reported that the gut microbiota of patients with schizophrenia exhibits significant imbalance, which is tightly linked to neuroinflammation, immune response, and neurotransmitter metabolism [104, 110]. In particular, the abnormal function of the D2 receptor is tightly linked to the positive symptoms of schizophrenia, and the overactivation of the D2 receptor is regarded as a key pathological feature of the disease [110].

Dopamine is one of the main neurotransmitters in schizophrenia, and it acts in the brain via dopamine receptors, in detail, the D2 receptor. The overactivation of the D2 receptor is regarded as one of the main causes of schizophrenia symptoms, in detail, positive symptoms (such as hallucinations and delusions) [110]. For instance, in patients with schizophrenia, the D2 receptor generally shows functional overactivation, which is linked to the disorder of dopaminergic signal transduction in certain brain regions (such as the striatum) [111]. Studies have also reported that the excessive release of dopamine (a key neurotransmitter) is tightly linked to the abnormal overactivation of the D2 receptor [112]. Pharmacological experiments have found that using drugs that can inhibit the D2 receptor can significantly change the symptoms of schizophrenia [110]. These findings support the dopamine hypothesis, that is, dopamine is central to the occurrence and symptom formation of schizophrenia.

Lately, mounting studies have centered on the potential that changes in gut microbiota may indirectly impact the pathogenesis of schizophrenia via the GBA [113, 114]. Gut microbiota imbalance is believed to impact brain function via multiple pathways, including changes in inflammatory factors and alterations in neurotransmitter metabolism [110, 111]. Exactly, gut microbiota imbalance may impact the function of the D2 receptor in the context of increasing inflammatory factors in the brain and modifying dopamine metabolism [78, 115]. For instance, changes in gut microbiota can promote the activation of the body’s immune system, causing the entry of inflammatory factors (such as IL-6 and TNF-α) into the brain [111]. These inflammatory factors can change neurotransmitter metabolism and trigger the functional abnormality of neurotransmitter receptors (such as the D2 receptor) [111]. Gut microbiota can impact the synthesis and metabolism of dopamine. For instance, some specific gut microbiota may promote the metabolism of tryptophan in the gut into 5-HT or other neurotransmitter precursors, hence indirectly impacting the level of dopamine in the brain [110]. These changes may promote the overactivation of the D2 receptor and result in the worsening of schizophrenia symptoms.

#### 3.4.3 The Interaction Between the D2 Receptor and Leaky Gut Syndrome

Leaky Gut Syndrome (LGS) refers to a condition where the intestinal barrier becomes compromised, allowing harmful substances, including toxins and pathogens, to leak into the bloodstream [116]. This condition is linked to chronic low-grade inflammation and has been implicated in a range of neuropsychiatric disorders, including anxiety, depression, and schizophrenia [117, 118]. The interaction between the gut microbiota, the D2 receptor, and LGS is of critical interest in understanding the pathogenesis of these disorders [62].

The D2 receptor, a key player in the dopamine system, modulates multiple brain functions, including mood, cognition, and behavior [119]. It is also involved in the regulation of gastrointestinal function, highlighting its role in the gut-brain axis [120]. Recent studies have reported that the D2 receptor’s dysfunction may contribute to emotional disturbances and cognitive impairments seen in conditions such as depression and anxiety [45, 121]. Additionally, D2 receptor activity in the gut influences the microbiota composition, which can, in turn, impact brain function and behavior [120].

The integrity of the intestinal barrier is crucial for maintaining homeostasis in the gut-brain axis [122]. In LGS, this barrier is weakened, eliciting an overactive immune response and chronic inflammation [123]. This inflammation can alter the composition of the gut microbiota, increasing the growth of pathogenic bacteria and reducing the abundance of beneficial bacteria [124]. The resulting dysbiosis may interrupt the dopaminergic system in the context of impacting the metabolism and signaling of dopamine, which plays a crucial role in mood regulation and behavior [52]. Research has reported that inflammatory cytokines produced as a result of LGS can impact the function of the D2 receptor, potentially altering its activity [125]. This impairment can result in dysregulated dopamine signaling, causing the development of mood disorders and exacerbating symptoms of other neuropsychiatric conditions [126]. Besides, the inflammatory response in the gut may result in the release of substances that compromise the blood-brain barrier, further increasing susceptibility to neurological dysfunction [127].

Studies have also demonstrated that gut microbiota imbalances resulting from LGS can impact the expression of the D2 receptor in both the gut and the brain [128, 129]. A gut microbiota dysbiosis, in particular, an enrichment in pro-inflammatory microbes, can result in the upregulation of inflammatory pathways that perturb the dopaminergic system [130]. This dysregulation causes alterations in dopamine secretion and receptor expression, triggering an increased risk of developing psychiatric disorders such as depression and anxiety [131]. Additionally, alterations in dopamine metabolism due to LGS-induced inflammation may impact the brain’s reward pathways, further triggering the emotional and behavioral dysregulation observed in these conditions [132]. The D2 receptor, therefore, acts as a vital mediator in this pathway, and its dysfunction in light of LGS may significantly impact the onset and progression of neuropsychiatric disorders [133].

Given the interaction between LGS, gut microbiota, and the D2 receptor, therapeutic strategies aimed at restoring intestinal barrier integrity and reducing gut inflammation may offer novel approaches for controlling mood disorders and other psychiatric conditions [134]. Probiotics, prebiotics, and anti-inflammatory treatments targeting the gut microbiota may offer potential benefits in restoring D2 receptor function and alleviating symptoms linked to LGS and neuropsychiatric disorders [135].

### 3.5 Limitations

This review delves into key aspects of the correlation between dopamine D2 receptors and gut microbiota, particularly in the context of emotion regulation and neuropsychiatric disorders. However, it must be acknowledged that there are several limitations. Although there is evidence to suggest that gut microbiota influences dopamine metabolism and D2 receptor expression, the exact molecular mechanisms are still not fully elucidated. The specific pathways through which gut microbiota modulates dopamine signaling require further exploration to gain a more comprehensive understanding of these interactions. Furthermore, most studies have focused on general microbiota changes in neurological and psychiatric disorders. Additionally, there is currently limited research on the regional changes in microbial composition and their exact effects on brain regions involved in emotion and behavior regulation, such as the prefrontal cortex and amygdala. Understanding how these regional differences impact dopamine receptor function is vital for future scholarly work. Over time, the dynamic interactions between gut microbiota and dopamine receptors have not been fully investigated. Most existing studies are cross-sectional, making it difficult to determine the causal relationship between gut dysbiosis and changes in dopamine receptor function. Longitudinal studies tracking changes in microbiota composition and D2 receptor expression over time will offer stronger evidence and insights for potential therapeutic windows. Many studies have focused on relatively homogeneous populations, limiting the generalizability of research results. Studying the impact of gut microbiota on D2 receptor function in different populations (including different genetic backgrounds, age groups, and comorbidities) will improve the generalizability of the results to a broader population. Comprehensive microbial community analysis is required. Although some studies have linked specific bacterial species (such as *Bacteroidetes* and *Prevotella*) to neurological and psychiatric symptoms, there is a lack of comprehensive microbiota analysis that includes a broader scope of bacterial species and their metabolites. Future scholarly work should focus on expanding microbial community analysis to discover other potentially important microbial communities and their collective implications for dopamine receptor signaling. In the context of addressing these limitations, future research can obtain a clearer understanding of the complex interactions between D2 receptors and gut microbiota, and how these interactions promote emotional regulation and neuropsychiatric disorders. This will also pave the way for developing more effective and personalized treatment strategies.

## 4. Conclusion

In conclusion, the dopamine D2 receptor is a key part of the GBA, playing a vital role in the regulation of emotion and behavior. The gut microbiota modulates the D2 receptor’s function, influencing mood regulation and contributing to the development of mood disorders. While the interaction between the D2 receptor and gut microbiota is increasingly recognized, the exact molecular mechanisms remain to be fully elucidated. Given the complexity of psychiatric disorders, it is likely that multiple mechanisms coexist, and further research is needed to delve into these interactions comprehensively. This could lay the groundwork for the development of more targeted and effective therapeutic strategies for mood disorders.

Future treatment strategies should focus on combining D2 receptor-targeted therapies with interventions aimed at modulating the gut microbiota, such as probiotics or dietary adjustments. This integrated approach holds the potential to enhance treatment efficacy and minimize side effects, offering a promising direction for the future management of mood disorders. Additionally, further research should explore the bidirectional interactions between the D2 receptor and gut microbiota, in particular, how these interactions impact neurotransmitter systems and emotion regulation. Advancing new targeted interventions that modulate both the D2 receptor and gut microbiota could offer innovative treatments, in particular, in cases where current drug therapies are limited due to side effects or insufficient efficacy. Ultimately, modulating the gut microbiota may offer a safer and more effective treatment approach, improving patient outcomes in managing mood disorders.
